# Weight loss before conception: A systematic literature review

**DOI:** 10.3402/fnr.v57i0.20522

**Published:** 2013-03-13

**Authors:** Elisabet Forsum, Anne Lise Brantsæter, Anna-Sigrid Olafsdottir, Sjurdur F. Olsen, Inga Thorsdottir

**Affiliations:** 1 Department of Clinical and Experimental Medicine, Linköping University, Linköping, Sweden; 2 Division of Environmental Medicine, Norwegian Institute of Public Health, Oslo, Norway; 3 Unit for Nutrition Research, Landspitali-University Hospital and University of Iceland, Reykjavik, Iceland; 4 Maternal Nutrition Group, Division of Epidemiology, Statens Serum Institute, Copenhagen, Denmark

**Keywords:** gestational diabetes, large-for-gestational-age-infants, systematic review, weight loss before pregnancy

## Abstract

The prevalence of overweight and obesity in women has increased during the last decades. This is a serious concern since a high BMI before conception is an independent risk factor for many adverse outcomes of pregnancy. Therefore, dietary counseling, intended to stimulate weight loss in overweight and obese women prior to conception has recently been recommended. However, dieting with the purpose to lose weight may involve health risks for mother and offspring. We conducted a systematic literature review to identify papers investigating the effects of weight loss due to dietary interventions before conception. The objective of this study is to assess the effect of weight loss prior to conception in overweight or obese women on a number of health-related outcomes in mother and offspring using studies published between January 2000 and December 2011. Our first literature search produced 486 citations and, based on predefined eligibility criteria, 58 were selected and ordered in full text. Two group members read each paper. Fifteen studies were selected for quality assessment and two of them were considered appropriate for inclusion in evidence tables. A complementary search identified 168 citations with four papers being ordered in full text. The two selected studies provided data for overweight and obese women. One showed a positive effect of weight loss before pregnancy on the risk of gestational diabetes and one demonstrated a reduced risk for large-for-gestational-age infants in women with a BMI above 25 who lost weight before pregnancy. No study investigated the effect of weight loss due to a dietary intervention before conception. There is a lack of studies on overweight and obese women investigating the effect of dietary-induced weight loss prior to conception on health-related variables in mother and offspring. Such studies are probably lacking since they are difficult to conduct. Therefore, alternative strategies to control the body weight of girls and women of reproductive age are needed.

The optimal body weight of pregnant women has been an issue of much debate over the years. It has long been recognized that underweight women tend to deliver small infants and a low birth weight is well known to be associated with increased mortality and morbidity in children (1). Recommendations regarding weight gain during pregnancy have also been given for a long time. For example, an American textbook on obstetrics (2) stated in 1966 that ‘Excessive weight gain in pregnancy is highly undesirable for several reasons; it is essential to curtail the increment in gain to 12.5 kg at most or preferably 6.8 kg’. However, this policy of severe weight restriction during pregnancy was challenged already in the 1960s when it was realized that such a restriction is associated with an increased risk for low birth weight infants and consequently with several health problems in the offspring (3).

In 1990, the Institute of Medicine (IOM) of the National Academy of Science in the United States published a report on weight gain during pregnancy where such recommendations were based on the prepregnant BMI of the woman (4). It was recommended that lean and underweight women gain more weight than normal weight women and those were in turn recommended to gain more weight than overweight or obese women. The IOM report of 1990 (4) thus implemented the important fact that the preconceptional nutritional situation of a woman is important for her nutritional requirements during pregnancy.

The prevalence of overweight and obesity in women of reproductive age has increased considerably during the last decades. For example, in Sweden this figure increased from 25 to 36% between 1992 and 2001 in pregnant women (5). This is a serious concern since a high BMI before pregnancy confers an increased risk of maternal and perinatal complications, including preeclampsia, gestational diabetes, caesarean delivery, large-for-gestational-age-infants, stillbirth and possibly an increased risk for overweight and obesity later in life in the offspring (5–7).

In 2009, IOM revisited their recommendations for pregnancy weight gain (Table 1) (6). The following statement was an important addition to their guidelines: ‘All women should start pregnancy with a healthy body weight’. A BMI within the range of normal BMI values (18.5–24.9) is considered to be a healthy body weight. This recommendation was made since ‘evidence from the literature is remarkably clear that prepregnancy BMI is an independent predictor of many adverse outcomes of pregnancy’ (6, 7). In fact, it currently appears that, for obese women, prepregnancy BMI is more associated with an increased risk of preeclampsia, gestational diabetes mellitus, and the delivery of a large-for-gestational-age (LGA) infant than is gestational weight gain (8). The recent IOM report emphasized that the full implementation of their guidelines would mean: ‘Offering preconceptional services, such as counseling on diet and physical activity as well as access to contraception, to all overweight or obese women to help them reach a healthy weight before conceiving’ (6).

**Table 1:** Weight gain during pregnancy as recommended by the Institute of Medicine 2009 (6)

BMI (kg/m^2^) before conception	Recommended weight gain (kg)
<18.5 (underweight)	12.5–18
18.5–24.9 (normal weight)	11.5–16
25.0–29.9 (overweight)	7–11.5
>30.0 (obesity)	5–9

A recently published systematic review demonstrated positive effects for mother and offspring as a result of weight reductions during pregnancy (9). Furthermore, the recent IOM report (6) presents evidence that weight loss prior to conception is associated with improved reproductive outcomes for obese women undergoing bariatric surgery (10, 11). However, no studies regarding the effect of weight loss as a result of interventions including dietary manipulations and implemented prior to conception in overweight and/or obese women were citied. This systematic literature review was conducted to identify published papers describing such studies.

## Research question

The original research question was: Is there scientific evidence for positive health effects of weight loss prior to conception for overweight and obese women? Potential outcomes: weight and length of infants at birth, macrosomia, length of gestation/prematurity, malformations, stillbirth, childhood obesity/BMI, obstetric risk, preeclampsia, postpartum weight retention, gestational diabetes mellitus, hypertension, postpartum depression, lactation and lactation duration, infant growth. The strategy used to find literature relevant for this research question is shown in Table 2. Two databases (PubMed and Swe Med) were searched.

**Table 2:** Search strategy for ‘Research Question’

(‘weight loss’[All Fields] OR ‘weight management’[All Fields] OR ‘weight counseling’[All Fields] OR ‘pre-pregnancy body mass index’[All Fields] OR ‘obesity intervention’[All Fields] OR ‘following bariatric surgery’[All Fields]) AND (‘pregnancy’[All Fields] OR ‘fertilization’[All Fields] OR ‘conception’[All Fields] OR ‘infertility’[All Fields] OR ‘fertility’[All Fields]) AND (‘infant, newborn’[All Fields] OR ‘fetal macrosomia’[All Fields] OR ‘pregnancy’[All Fields] OR ‘congenital abnormalities’[All Fields] OR ‘stillbirth’[All Fields] OR ‘pre-eclampsia’[All Fields] OR ‘diabetes, gestational’[All Fields] OR ‘hypertension, pregnancy-induced’[All Fields] OR ‘depression, postpartum’[All Fields] OR ‘lactation’[All Fields] OR ‘breast feeding’[All Fields] OR ‘abortion, spontaneous’[All Fields] OR ‘bariatrics’[All Fields] OR ‘infant, low birth weight’[All Fields] OR ‘infant, very low birth weight’[All Fields] OR ‘Obstetric Risk’[All Fields] OR ‘Weight Management’[All Fields] OR ‘Obesity Intervention’[All Fields]) AND (‘2000/01/01’[PDat]: ‘2010/07/15’[PDat]) AND (‘Humans’[MH] OR Human*[TIAB])

## Literature search

**Fig. 1:**
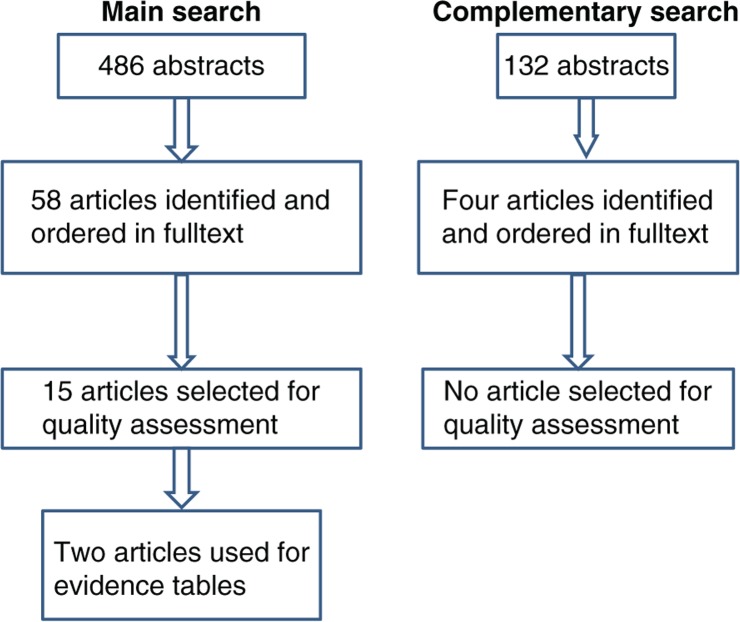
Description of literature search, including main and complementary search.

The literature search is described in Fig. 1. The main search was conducted in November 2010, covering articles published between January 1, 2000, and July 15, 2010, and identifying 486 abstracts. These articles were read by three members of the pregnancy-and-lactation-group. EF read all abstracts while IT and AS each read 50% of the abstracts. Thus, two persons read all abstracts. Abstracts were identified according to the following criteria: obesity and overweight before pregnancy (or between pregnancies) and change in body weight before pregnancy and any kind of health-related outcome including intervention trials (>1 month) but excluding weight loss by surgery. In this way, 58 articles (5, 12–68) were identified and ordered in full text. Two members of the group read each of the 58 articles and if at least one member considered an article appropriate, it was selected for quality assessment. Review articles were excluded but otherwise inclusion criteria were the same as those used to identify abstracts. Reasons for excluding 43 of the articles (12–54) are shown in the appendix. Thus, this procedure resulted in 15 articles for quality assessment (5, 55–68).

## Final selection of articles and quality assessment

The 15 articles were distributed between the five group members. Each member read 5–7 articles and two members, who also carried out the quality assessment of their articles, read all articles. Among the 15 selected articles 11 (56, 58, 59, 61–68) were found not to be relevant while four (5, 55, 57, 60) were considered relevant and of sufficient quality for inclusion in evidence tables. However, one of those (57) investigated the relationship between a reduction in BMI and a preterm birth but involved mainly low-to-normal-weight women and was thus not considered relevant for our review. Another (55) was a review emphasizing the lack of relevant studies for our particular research question. Thus, two studies, Villamor and Cnattingius (5) and Glazer et al. (60) were used in evidence tables providing data for two outcomes, i.e. risk of gestational diabetes and risk of LGA infants (Table 3). To assess and rate the quality of the included studies, we applied a three-category (A–B–C) grading system based on the NNR AMSTAR quality assessment tool (QAT).

**Table 3:** Table for evidence grading: risk of gestational diabetes and risk of delivering a large-for-gestational-age infant

Reference details	Glazer N et al. (60), USA	Villamor and Cnattingius (5), Sweden	Villamor and Cnattingius (5), Sweden
Study design	Prospective cohort study	Prospective cohort study	Prospective cohort study
Population/subject characteristics	Obese women (heavier than 200 lbs or 90.7 kg) of mixed ethnicity with 2 singleton births who were nondiabetic at the first pregnancy	Women in Sweden giving birth to two consecutive singletons between 1992 and 2001	Women in Sweden giving birth to two consecutive singletons between 1992 and 2001
No of subjects analysed	4,012	313 (from 151,025) for risk of gestational diabetes	2,350 (from 151,025) for risk of delivering a large-for-gestational-age infant
Outcome measures	Risk of gestational diabetes at the second pregnancy	Risk of gestational diabetes at the second pregnancy	Risk of delivering a large-for gestational-age (LGA) infant at the second pregnancy
Exposure	Prepregnancy weight at an index pregnancy minus the corresponding weight at the previous pregnancy	Difference between the two pregnancies with respect to BMI recorded at the first antenatal visit	Difference between the two pregnancies with respect to BMI recorded at the first antenatal visit
Follow-up period, drop-out rate	Nine-months follow-up, no drop-outs	Nine-months follow-up, no drop-outs	Nine-months follow-up, no drop-outs
Dietary assessment method	No dietary assessment	No dietary assessment	No dietary assessment
Results	Women who lost at least 10 lbs (4.54 kg) between pregnancies had a decreased risk of gestational diabetes relative to women who lost less weight during this period (relative risk=0.63, 95% CI, 0.38–1.02)	Overweight and obese women who decreased their BMI more than one unit between pregnancies had no significant reduction in the risk of gestational diabetes (OR 0.96, 95% CI, 0.66–1.37)	Overweight and obese women who decreased their BMI more than one BMI-unit between pregnancies had a significant reduction in the risk of giving birth to a LGA-infant (OR 0.82, 95% CI, 0.72–0.95)
Confounders adjusted for	Age and weight gain during each pregnancy	Height, interpregnancy interval, age, country of origin, years of education, year of delivery and smoking	Height, interpregnancy interval, age, country of origin, years of education, year of delivery and smoking
Study quality and relevance	Study quality: B. The study is not quite relevant since there is no information that the women received dietary advice and we do not know why they lost weight	Study quality: A. The study is not quite relevant since there is no information that the women received dietary advice and we do not know why they lost weight	Study quality: A. The study is not quite relevant since there is no information that the women received dietary advice and we do not know why they lost weight

## Complementary search

At the end of January 2012, a complementary search (Fig. 1) was conducted covering the period between July 15, 2010, and the end of December 2011. The same search string and databases were used as in the main search. The complementary search resulted in 132 abstracts. These were read by two members of the group (EF and IT) and resulted in four articles (69–72) being ordered in full text. None of them were selected for QAT.

## Results

Glazer et al. (60) provided evidence for a positive effect of weight loss (at least 10 lbs or 4.54 kg) between pregnancies on the risk of gestational diabetes during the subsequent pregnancy. Such an effect was not demonstrated by Villamor and Cnattingius (5) possibly because the women in their study weighed less and lost less weight than the women in the study by Glazer et al. (60) (Table 3). It is of interest to note, however, that the former study (5) demonstrated clearly that weight gain between pregnancies is associated with adverse health effects in mothers as well as in infants also when it occurs in normal-weight women. Furthermore, the study by Ehrlich et al. (70) confirms the findings by Glazer et al. (60) that weight loss between pregnancies, in obese and overweight women, has a positive effect on the risk for gestational diabetes in the subsequent pregnancy. Furthermore, the study by Villamor and Cnattingius (5) demonstrated a reduced risk for LGA infants in women with a BMI above 25 who lost weight equivalent to at least one BMI-unit before their next pregnancy (Table 3).

## Discussion

As part of the review process, a referee alerted us about a paper by Getahun et al. (73) where changes, increases as well as decreases, in BMI during the first two pregnancies of more than 700,000 American women were analyzed in relation to LGA-births. This paper was not captured by our research question, probably since it was not presented as a paper focusing on weight loss. However, a decrease in BMI must be due to a loss of body weight. Getahun et al. (73) reported that obese women were at an increased risk for delivering LGA-infants. Furthermore, although the risk for delivering such an infant was attenuated if an obese woman lost weight between the two pregnancies, the risk was still higher than for normal weight women who maintained their body weight between their first two pregnancies.

Our research question did not include a statement requiring that weight loss should be the result of a dietary intervention. Nevertheless, it is evident from our review that studies regarding preconceptional dietary-based interventions aiming at weight reduction in overweight and obese women are currently lacking. Our literature search, including our complementary search, clearly shows that many women would benefit substantially from such a weight loss. Probable positive effects include improved reproductive outcome and improved health of mothers, for example reduced preeclampsia (71), as well as improved health of offspring. However, it is conceivable that preconceptional dietary-based interventions aiming at weight reduction in overweight and obese women also may have harmful effects, for example risks of nutritional deficiencies (i.e. iron or folate) or disorders related to eating behavior. Another concern is pointed out by Zhang et al. (74) in a recent paper where the authors discuss evidence indicating that undernutrition as well as overnutrition, imposed during the periconceptional period, may both affect the offspring negatively. Thus, it was stressed in their paper that ‘it is important to ensure that any dietary restriction interventions recommended for overweight and obese mothers are evidence-based to allow for an effective weighing up of the potential metabolic benefits and costs for the offspring’. The present paper shows that evidence-based strategies regarding how dietary interventions before conception should be carried out to be successful whilst simultaneously avoiding potentially harmful effects, are currently lacking. Although urgently needed such studies seem to be very difficult to carry out. The obvious reason for the lack of scientific evidence is a lack of data since recruiting women before conception is associated with practical problems. An alternative approach to the problem of overweight and obesity in reproductive women could be to develop public health strategies where serious efforts are made to counteract overweight and obesity in girls and young women. Additional efforts helping women to gain weight during pregnancy according to recommendations and to lose weight after delivery would be important parts of such a strategy. It should be emphasized, however, that efforts to control body weight should not occur at the prize of a nutritionally adequate dietary intake. Achieving these goals represents a difficult task but a task of considerable public health importance.

## Appendix

**Table:** Papers ordered in full text but not included in the systematic literature review. The reason for exclusion is also given.

Papers excluded	Reason for exclusion
Anonymous (12)	Not relevant for research question
Anonymous (13)	Not relevant for research question
Anonymous (14)	Not relevant for research question
Barger et al. (15)	Not relevant for research question
Bellver et al. (16)	Not relevant for research question
Bitsko et al. (17)	Focus on safety of weight loss products, Not relevant
Bo et al. (18)	Study of maternal low birth weight, Not relevant
Caughey et al. (19)	Not a research paper, Not relevant
Coitinho et al. (20)	Not relevant for research question
Frederick et al. (21)	Deals with weight gain, not weight loss, Not relevant for research question
Galtier et al. (22)	Not relevant for research question
Gunderson et al. (23)	Not relevant for research question
Haugen et al. (24)	Not relevant for research question
Hegaard et al. (25)	Not relevant for research question
Jevitt (26)	Not relevant for research question
Johnson et al. (27)	Not relevant for research question
Jones et al. (28)	Not relevant for research question
Keller et al. (29)	Not relevant for research question
Kuchenbecker et al. (30)	Review, Not main topic, Not relevant
Kuhlmann et al. (31)	Wrong topic, Not relevant
Lagiou et al. (32)	Wrong topic, Not relevant for research question
Le Goff et al. (33)	Review
Lederman et al. (34)	Wrong topic, Not to the point, Not relevant for research question
Ly et al. (35)	Wrong topic, Not relevant
Maloni et al. (36)	Wrong topic, Not relevant
McGuire et al. (37)	Wrong topic, Not relevant
Metwally et al. (38)	Wrong topic, Not relevant
Morisset et al. (39)	Not relevant for research question
Nelson et al. (40)	No appropriate outcome, Review
Ostbye et al. (41)	Not appropriate for research question
Pandey et al. (42)	No appropriate outcome, Review
Rah et al. (43)	Not appropriate for research question
Rooney et al. (44)	Not appropriate for research question
Rooney et al. (45)	Not appropriate for research question, Not relevant
Saleh et al. (46)	Not appropriate for research question, Not relevant
Seli et al. (47)	Not appropriate for research question, Review, Not relevant
Tema (48)	About low birth weight, not relevant for research question
Turhan et al. (49)	Not relevant for research question
Walker et al. (50)	Not relevant for research question
Vallianatos et al. (51)	About weight gain, not weight loss, Not relevant
Weissgerber et al. (52)	Not relevant for research question, Review
Winkvist et al. (53)	Not relevant for research question, Review
Yogev et al. (54)	Not relevant for research question
